# Supplementary material for: Are carbon emissions trading and green financial instruments synergistic? -Comprehensive quantitative research based on content analysis
Source: PLoS One. 2024 Mar 7;19(3):e0298601. doi: 10.1371/journal.pone.0298601 (PMC10919601; doi:10.1371/journal.pone.0298601)
Supplement: S1 Data — (ZIP) [file pone.0298601.s001.zip › Data/地级市Z_F工作报告.zip!/║┌┴·╜¡/║┌║╙╩╨/└┤╫╘╣1⁄2╓┌║┼í╛┬φ┐╦╩2╛▌═°í┐_2019.docx]

各位代表：

现在，我代表市人民政府向大会作工作报告，请予审议，并请列席会议的同志提出宝贵意见。

一、2018年工作回顾

2018年是我国改革开放40周年，我们以习近平新时代中国特色社会主义思想为指导，坚决贯彻落实习近平总书记在深入推进东北振兴座谈会上的重要讲话和考察黑龙江的重要指示精神，紧扣省委、省政府和市委各项决策部署，积极应对经济下行压力加大的严峻挑战，坚持抓投资、抓项目、抓招商、抓环境、抓改革开放创新、抓解放思想，经济社会实现平稳健康发展。全市地区生产总值增长7%，固定资产投资增长7.1%，社会消费品零售总额增长8.3%，公共财政收入增长7%，对外贸易进出口总值增长19.1%，城镇、农村常住居民人均可支配收入分别增长6.96%和9%，预计万元地区生产总值综合能耗下降2%。

——现代农业提质增效。坚持向特色要发展，向质量要效益，农业由增产增效向提质增效加快变革。创建农业绿色发展示范区成效显著，全面铺开由三年提速到两年，建立生产记录、质量追溯等制度，构建绿色种植技术体系，十大绿色特色功能性农产品生产基地面积1184.9万亩，新增绿色有机食品认证数116个、地理标志农产品1个，黑河被农业农村部确定为全国唯一的绿色高质高效示范市，嫩江被财政部、粮食和物资储备局确定为中国好粮油大豆示范县，瑷珲镇成为国家农业产业强镇。加快发展现代养殖业，规模化养殖场达到317个，名特优水产养殖面积达到8.9万亩，北安和嫩江被列入全省畜牧重点县，非洲猪瘟等动物疾病防控工作扎实推进。实施林业产业发展三年行动计划，建立省级大果沙棘工程技术研究中心，林果、北药种植规模不断扩大。强化农产品营销，在发达地区设立农产品特产综合馆和绿色食品展示店，加大嫩江“十六膳”、五大连池“冷矿泉暖米”、五大连池风景区“矿泉鱼”、逊克“北纬49”等品牌宣传力度，黑河大豆网上线运营，全市线上农产品销售率达到10.5%。推进农业农村重点领域改革，土地承包经营权证发放和村级集体资产清产核资工作全面完成，国有林场改革通过省级评估验收。

——工业经济步伐加快。坚持“抓大、扶小、解困”，全力扩大总量、盘活存量、引进增量、提升质量，规模以上工业增加值增长16%，创五年来最高增幅。合力攻坚产业项目，全市重点产业项目开复工73项、完成投资72亿元，其中省重点开复工18项、完成投资34亿元，开复工率和投资完成率均达到90%以上；多宝山铜矿二期开工，东安岩金矿、农耕大豆深加工等33个重点产业项目完成全部建设任务，建成投产率达到44%，为三年来最好水平。加强与珠海对口合作，在农业、旅游、金融、航线等方面取得积极进展。强化精准招商，坚持大招商、招大商，玉米燃料乙醇、天之草汉麻纤维加工等31个超亿元项目落地，实际利用省外资金和外资分别增长6%、7%。谋划推进牵动性强的替代项目，高纯荧光粉、冰花罐装啤酒等项目开工建设，盘活龙江化工10万吨电石、嫩江春酿酒等停产半停产企业22家。深入实施中小企业成长工程，新增规模以上工业企业5家；建成各类科技企业孵化服务机构9家，在孵科技型小微企业和创新团队143个，新培育和创建科技型中小企业73家，新认定高新技术企业3家，自主创新活力和综合竞争力不断增强。

——对俄合作乘势而上。坚持抢抓中俄地方合作交流战略机遇，以互联互通为牵动，推动黑河由“地理大通道”向“经贸大通道”转变。外贸扭转连续四年下滑态势，黑河口岸货运量58万吨，增长51%，创开关以来历史最高；在俄农业种植面积134万亩，鲜蛋产量占阿穆尔州40%，粮食进口占全省1/3。黑龙江公路大桥加快建设，中俄东线天然气管道两条穿江隧道贯通，跨江空中索道我方具备开工条件，嫩江墨尔根通用机场通航。完善对外开放平台，桥头区基础设施建设启动，口岸联检设施开工，保税物流中心(B型)海关监管区主体完工，境外园区累计投资超亿美元，跨境物流枢纽项目纳入国家布局，油品储运、锑矿开采项目正在进行开工准备。成功举办第九届中俄文化大集、中俄边境城市展览会等重大活动，中俄界江国际冰球友谊赛成为两国交往机制化项目，中俄青少年科普文化交流成为全省科普专项品牌活动，中俄国际综合艺术团助演第四届东方经济论坛，友城布拉戈维申斯克被中国友协授予“对华友好城市交流合作奖”，是全省唯一获奖的地级市友城。

——新的动能加速集聚。坚持发挥整体生态优势，加快景区景点建设，实施瑷珲乡野公园、北安富强田园综合体等重点旅游项目16个，黑龙江游船瑷珲航线通航，逊克边境游线路开通。汇聚全市智慧和力量参加央视《魅力中国城》竞演并摘取最高奖“十佳魅力城市”称号，“黑河·我的极致远征”跨境自驾游获年度魅力主题线路奖，央视《东西南北贺新春》《大手牵小手》等品牌节目在黑河录制，举办了旅游招商推介会、全省首届古驿路历史文化与旅游发展论坛，建立了“胡焕庸线”院士工作站。五大连池风景区荣获全省唯一的第三届中国质量奖提名奖，入选国家5A级景区50强，旅游品牌价值居全省首位。全市接待国内外旅游者增长18.9%、突破千万人次大关，旅游收入增长22.5%、达到百亿元关口。扩大寒区试车优势，深化与国内外寒区试验场、科研院所交流合作，中国寒地试车产业园加快推进，成功举办瑷珲寒地国际汽车巡游和试车产业发展论坛，被中汽协授予全国唯一的“中国汽车寒区试验基地”称号，试验企业、车辆、人员分别增长10.8%、24%、64%，均创历史新高。大力发展电子商务，全市电商园区达到5个，网络交易额12亿元、增长20%，合作区跨境电商产业园入驻企业超百家，在全省首家引入京东云仓，布市海外仓建成运营，境内外供应链进一步完善。

——城乡建设扎实推进。坚持以创建全国文明城市为契机，提升城市品位，完善城市功能，黑河通过国家园林城市复检，五大连池成功创建国家卫生城市，嫩江撤县设市通过民政部专家评审。城区防洪工程和四岛防护工程全面完工，新建在水一方广场，沿江公园完成改造，体育公园投入使用；启动市区餐厨垃圾集中收运，生活垃圾处理厂完成验收；拆除违法建筑172个，石材大市场完成整体拆迁。全市开工改造棚户区3718户、27.3万平方米，改造市区老旧楼房103栋。以高度政治自觉推进中央环保督查问题整改，57件信访案件全部办结，“大棚房”专项清理整治95个问题全部完成整改。全力打好原生态、蓝天、碧水、净土、美丽乡村“五场保卫战”，被评为省级生态市，造林绿化6.06万亩、森林抚育25.6万亩，连续25个防期未发生重特大森林火灾、5个防期零火灾；全市淘汰建成区燃煤小锅炉59台，秸秆实现全域全时段禁烧，空气质量达标天数稳居全省前列；严格落实河湖长制，全省最严格水资源管理制度考核名列前茅；在全省首倡并实行农药、除草剂、饲料添加剂使用负面清单管理和市场准入制度；全面开展农村人居环境整治行动，实施“三清理一绿化”专项整治，建设农村公路138.8公里，新建配建室内厕所355座，507个村建立了保洁员队伍，农村环境更加整洁优美。

——民生福祉持续改善。坚持以人民为中心的发展思想，满足群众多样化需求，让群众生活更舒心安心顺心。财政用于民生领域支出186.4亿元，占一般公共预算支出88.5%，146项民生项目建成使用。稳步提高群众生活水平，住户存款595亿元，比年初增长12.1%。脱贫攻坚取得重大进展，建档立卡贫困户危房改造任务和贫困村、贫困人口饮水安全工程全部完成，41个贫困村、8011名贫困人口实现脱贫，孙吴省级贫困县成功摘帽，全市贫困发生率由年初的1.48%降至0.31%。就业形势持续向好，城镇登记失业率控制在3.57%以内，比全省低0.42个百分点。引进高层次人才15人，启动撤地建市以来规模最大的事业单位公开招聘。新建改扩建公办幼儿园10所，义务教育标准化学校全部达标，高考成绩实现“七连增”，省部共建国家现代农村职业教育改革工作高质量推进。市疾控中心改扩建、五大连池人民医院康复中心综合楼完工，北安被确定为全省公立医院综合改革示范县，孙吴“智慧医疗”试点项目投入使用，爱辉区成功创建国家健康促进示范区。文化事业繁荣发展，黑河被授予全民阅读示范城市，知青博物馆挂牌为国家方志馆知青分馆。在省第十四届运动会上金牌和奖牌数量创历史最好成绩，黑河全民健身中心、五大连池文体中心投入使用，北安冰球馆完成主体工程，逊克全民健身中心完工。完成城乡居民医疗保险整合，社会保险基本实现法定人群全覆盖，低保、特困供养人员和医疗救助标准全面提高。安全生产形势总体稳定，未发生较大以上安全生产事故。严格落实信访责任制，妥善解决了52个项目、1.5万余户居民不动产权证办理难题，中央第六巡视组移交信访事项全部办结。深入推进“平安黑河”建设，“扫黑除恶”专项斗争取得阶段性明显成效。加强国防后备力量建设，筑牢边境安全屏障，公共安全形势持续平稳。老区、侨务、妇女儿童、民族宗教、人防、防震减灾、档案方志等工作取得新成绩。

一年来，我们坚持党的全面领导，坚决在思想上政治上行动上同以习近平同志为核心的党中央保持高度一致。自觉接受市委领导，主动接受人大依法监督和政协民主监督，48件人大代表建议、152件政协委员提案全部办结。深入开展解放思想推动高质量发展大讨论，强力推进政府系统作风建设专项整治，大力倡导走上去、走出去、走下去，提振精神状态，增强履职本领，干事创业的氛围更加浓厚。扎实开展“法治建设年”活动，完善市政府重大决策制度，实施社会稳定风险评估26项，重大决策合法性审查率100%，市县两级政府全部建立法律顾问制度。全力优化营商环境，“一网一门一次”改革扎实推进，667项市级政务服务事项实现“跑一次、不见面、网上办”，41.1%市级审批事项办理时限压缩一半，网上审批效率提高56.5%，营商环境总体评价满意度87.9%，高于全省5.5个百分点。严格履行“一岗双责”，强化审计监督，政府系统党风廉政建设进一步加强。

各位代表，过去的一年，全市经济社会发展取得了积极进展，这是省委、省政府和市委正确领导的结果，是市人大、市政协和社会各界鼎力支持的结果，是全市人民团结一致、共同奋斗的结果。在此，我代表市人民政府，向全市各级人大代表、政协委员和各民主党派、工商联、无党派人士、人民团体，向关心支持地方工作的中省直单位和人民解放军、武警官兵、消防救援队伍指战员，向辛勤工作在各行各业的全市人民，表示衷心的感谢并致以崇高的敬意！

在看到成绩的同时，我们也清醒认识到面临的困难和问题：经济总量小，发展速度慢，大项目数量不多，发展支撑力不强；三次产业融合程度低，特别是产业链短、价值链低的问题突出，发展质量不高；人才引进难度大、人才外流多，高层次人才、新兴产业人才缺乏；基础设施和民生事业欠账较多，一些突出短板还没有补齐，与人民群众对美好生活的期盼还有较大差距；干部思想解放、观念更新迫在眉睫，“旧、怕、平、懒”等现象依然存在，营商环境亟待优化。这些问题既是压力，也是潜力，更是动力，我们一定坚持问题导向，强化责任担当，敢于攻坚克难，以革故鼎新的勇气，努力把各项事业推向前进。

二、2019年工作安排

2019年是新中国成立70周年，是全面建成小康社会关键之年。习近平总书记深入东北三省考察，亲自谋划推动东北振兴，即将出台新一轮支持东北振兴政策，稳边固边、兴边富民利好措施加速落地，黑河正处于政策叠加的机遇期；中俄全面战略协作伙伴关系步入新阶段，黑龙江公路大桥、中俄东线天然气管道、跨江空中索道将再次聚焦世界目光，边缘劣势变地缘优势，偏远地域变开放前沿，黑河正处于趋势看好的关键期；拥有极具品牌价值的农业绿色发展示范区、极具后发潜力的矿产资源、极具稀缺性的生态环境、极具竞争力的特色旅游名片，发展要素齐全，优势加速聚集，黑河正处于潜力释放的腾飞期。我们要坚定信心、借势发力，加快把发展机遇转化为发展成效，在推动全面振兴全方位振兴的伟大征程中展现新气象、体现新担当、实现新作为，积极推进经济社会实现跨越式发展。

做好今年政府工作，要按照市委部署，坚持以习近平新时代中国特色社会主义思想、习近平总书记在深入推进东北振兴座谈会上的重要讲话和考察黑龙江的重要指示精神为指引，全面贯彻落实党的十九大和中央、省委经济工作会议精神，遵循巩固、增强、提升、畅通“八字方针”，抓住历史性发展机遇，坚持高质量发展方向，围绕全市地区生产总值到2021年突破千亿元目标，以产业项目建设提速增效为核心，一二三产业共同发力，开发与开放并举，生态建设与经济发展同频，推进全面振兴登上一个新台阶，加快实现总量倍增、位次前移、殷实和谐，给人民群众带来更稳更好的幸福，逐步将黑河打造成我省向北开发开放的重要经济增长极，以优异成绩庆祝中华人民共和国成立70周年。主要预期目标是：全市地区生产总值增长7%以上，固定资产投资增长10%，社会消费品零售总额增长8%，公共财政收入增长8%，对外贸易进出口总值增长6%，城镇、农村常住居民人均可支配收入均增长7%，万元地区生产总值综合能耗下降2%。

各位代表，今后三年是实现市六次党代会确定奋斗目标的持续攻坚阶段，我们要把发展作为解决一切问题的“总钥匙”，深刻把握解放思想的丰富内涵，坚决落实高质量发展的本质要求，充分发挥绩效考核的激励引导作用，把谋划变规划、规划变计划、计划变现实，跑出规模扩张、结构优化、质量提升的加速度。一年强基础，促进项目集中、企业集聚、产业集群、要素集约，形成多点支撑、多业并举、多元发展的新格局，地区生产总值保持中高速增长，城乡居民收入与地区生产总值同步提高，脱贫攻坚取得决定性胜利，发展的四梁八柱更加牢固。两年促提升，传统优势产业向中高端迈进、战略性新兴产业和现代服务业向新增长领域拓展，系统形成新的经济增长点，综合实力稳居全省第二方阵前列，成功跻身全国文明城市，与全国全省同步全面建成小康社会。三年大变样，高质量发展的“五大目标”如期实现，“十大新体系”全面建立，力争地区生产总值迈进千亿元行列，城乡居民收入增幅跑赢地区生产总值，形成营商环境好、创新能力强、生态环境优、发展活力足的跨越式发展新局面。

2019年，我们要坚持发展高目标、工作高标准、结果高成效，坚持创新驱动、投资拉动、开放带动，统筹兼顾、突出重点，着力抓好以下工作：

(一)坚定实施产业项目攻坚行动，以高质量项目支撑高质量发展。深入实施新一轮产业项目三年行动，扎实开展“准备之冬、动工之春、大干之夏、成果之秋”活动，把一切优势资源聚焦到产业项目和招商引资上，实现经济工作项目化、项目工作具体化，不断增强发展内生动力。围绕扩大投资抓项目。全市安排重点产业项目80项，年度计划投资85亿元。坚持把矿业经济打造成支柱产业，全面落实与省地矿局战略合作协议，加快重点成矿区地质勘查，重点推进黑花山铜钼矿、三五八高地金矿、二道坎银矿等项目勘查，大平南山、永新、孟德河岩金矿等项目转采建矿，加快建设多宝山铜矿二期、铜山铜矿采矿等项目，确保翠宏山铁多金属矿、振兴煤矿等项目投产达效。坚持把闲置资产变成优质资源，做大做强以电熔结晶镁、氮化硅等为代表的替代项目，促进三精制药等企业恢复生产，力争盘活更多的停产半停产企业。强化服务保障，成立市县两级产业项目工作专班，推动项目快开工、快投产、快达效。围绕园区创新抓项目。明确园区功能定位，科学确定主导产业、空间规划，实现特色化、专业化、集约化。实施园区优化整合，推进新“九通一平”，加快产业项目和发展要素向园区集聚。加大清理处置低效利用土地和闲置土地力度，腾出更多空间用于高质量项目。探索“政府+公司化”管理模式，推动市场化运行、企业化管理、绩效化考核，引入龙头企业运营产业园区、“园中园”，与珠海合作共建“飞地园区”。拓展黑河进出口加工产业园俄电使用范围，谋划发展高载能产业，建设俄能源储备加工基地。围绕精准招商抓项目。突出农林、矿产、旅游康养、战略新兴等优势产业，引进投资额5000万元以上项目100个，其中超亿元项目50个，实际利用省外资金和外资均增长6%；各县(市、区)总投资3000万元以上项目全部纳入市项目储备库。全面提升招商队伍政策掌握能力、产业研究能力、市场分析能力、优势挖掘能力、优质服务能力，打造专业化高素质的招商干部队伍。创新招商方式，聚焦重点地区、重点产业和重点企业，做好空间、资金和项目准备，采取小团组、市场化等方式精准招商，深入开展对口合作招商，成立专班对接省产业投资集团，合作推进项目落地。建立“专班+园区”“目标+考核”招商引资新机制，实行全过程督查反馈，提高招商实效。围绕县域经济抓项目。树立全市一盘棋思想，根据资源、区位和产业基础，突出差异化、特色化，持续深挖潜力，精干主导产业，强化项目支撑，努力形成特色鲜明、优势互补的县域经济发展新格局。北安、嫩江要当好县域经济排头兵，助推全市经济快速发展。

(二)全面铺开农业绿色发展示范区创建工作，打牢乡村振兴坚实基础。以实施乡村振兴战略为统领，以增加农民收入为核心，以产业兴旺为第一任务和第一动力，坚定不移走绿色兴农、质量兴农之路，加快创建农业绿色发展示范区，发展壮大村集体经济，提高乡村治理能力和治理水平现代化，让农业更强、农村更美、农民更富。扩大绿色种植养殖规模。全面落实粮食安全行政首长责任制，按照特色要特、特色要优标准，调优调绿种植结构，农作物播种面积稳定在1900万亩以上，十大绿色特色功能性农产品生产基地达到1200万亩。坚持藏粮于地、藏粮于技，构建以大豆为核心的科学轮作体系，全面执行绿色有机生产技术标准体系，推进标准化生产全覆盖。加快发展林产经济，新增北药、林果种植面积10万亩以上。做大高档食用菌产业，扩大沙棘木耳、猴头菇等特色产品规模，用“小蘑菇”撑起富民大产业。大力发展“两牛一猪一马”，安格斯肉牛存栏超万头，黑河马达到1.8万匹，全市规模养殖场和渔业养殖全部达到绿色标准。严格农产品质量安全监管，做好非洲猪瘟等重大疫病防控，推进市质量技术检验检测中心建设，提高农产品监管认证能力，绿色有机食品认证面积达到600万亩，认证数量达到300个，让黑河的优质粮、放心肉更多装进“中国饭碗”。大力推进农林产品精深加工。围绕“两头两尾”，支持战略性、支撑性的大型加工企业发展，鼓励小型化、个性化绿色加工，推动农林产品加工差异化发展。大豆重点推进科迪大豆、农耕大豆等项目，提高保健、时尚、休闲食品研发加工能力，延伸产业链，提升价值链，努力把大豆转化为终端食品；玉米重点推进燃料乙醇项目，积极向淀粉、菲汀、味精等下游产品延伸；汉麻要扩大麻皮麻茎纤维生产加工规模，重点开发制药、食品、建材等产品，打造从种植优势到产业优势的全产业链；北药和小浆果重点推进水飞蓟深加工、中药饮片、保健饮品等项目，提升产品附加值；增强生猪、安格斯肉牛、黑河马、乳制品等畜产品加工能力，促进过腹增值、过腹增收。强化农产品市场营销。组建市绿色农业发展有限公司，集中打造黑河绿色有机功能性物产区域公共品牌。整合县级农产品电商平台，组建统一的农产品销售网络，打造网上超市。完善绿色食品体验店、专营店功能，扩大高端市场份额。举起非转基因大豆大旗，力争孙吴等5个交易库实现实质性交割，建设国家级非转基因大豆交易中心和集散中心。加大品牌推广力度，在全市主要出入口设立农业绿色发展示范区宣传牌，发动干部群众利用微信平台宣传推介，协调通信运营商集中推送广告，在国内主流媒体开设专栏专题节目，联合珠海、“魅力中国城·城市联盟”会员城市开展宣传营销，擦亮黑河绿色有机功能性物产“金字招牌”。培育壮大新型生产经营主体。发挥市绿色有机农业产业贷款风险补偿基金作用，支持专业合作社和致富能人领办创办加工企业。结合三权分置改革，发展农村土地、集体资产股份合作，让资源变资产、资金变股金、农民变股东。推动有条件的合作社组建联社，鼓励合作社实行股份制改造，加快实现企业化经营。加大农村实用人才培养力度，打造新型职业农民队伍。建设美丽宜居乡村。全面启动农村人居环境分类分标准推进工作，深入开展“三清理一绿化”专项行动，加快拆除1.4万个长期无人居住农村危旧房，改造农村低保户、分散供养特困人员、贫困残疾人家庭三类人员危房1477户，建设农村饮水安全巩固提升工程105处、受益7.42万人，完成167个行政村绿化任务，提高垃圾处理和污水治理能力，解决农村环境脏乱差等突出问题。积极推进“厕所革命”，既顺应村情民意，又切合北方实际，不搞一刀切。继续推进150个美丽宜居乡村建设，带动全市农村人居环境整体提升，让每一个村庄都各美其美、幸福宜居。

(三)全力构建对俄开放合作高地，加速通道经济向口岸经济转型升级。深入研究“大桥经济”形成的新比较优势和发展题材，深度融入共建“一带一路”，以跨境互联互通基础设施为载体，以进出口加工和服务贸易为重点，通过经济要素跨境流动带动经济活动，实现内外平台互动、开放政策呼应、人文合作互融。完善互联互通网络。黑龙江公路大桥建成通车，口岸联检设施完成安装调试，中俄东线天然气管道建成通气，推动中石油在黑河设立公司、中石化在黑河设立后勤保障基地，跨江空中索道开工。加快跨境物流枢纽区建设，探索组建中俄跨境物流运输公司，提升通关便利化水平，建设陆上边境口岸型国家物流枢纽承载城市。加快北黑铁路改造升级和北五铁路前期工作，推进嫩双公路嫩江段、逊孙公路逊克口岸段建设，开工丹阿公路黑河—卧牛湖段、五汤公路沾河—大平台段扩建工程。建设瑷珲机场廊桥，开通珠海—郑州—黑河航线，推进逊克通用机场前期，启动五大连池德都机场候机室二期，加密嫩江—哈尔滨航线，开通嫩江—漠河航线。搭建开放合作平台。争取重点开发开放试验区方案获批，推动设立边境旅游试验区，探索与布市共建“跨国特区”，积极申报国家级自贸区，建设省兴边富民示范城市，打造中俄边境地区政策最优、机制最活的开放区域。加快黑龙江公路大桥桥头区开发建设，多渠道筹资完善基础设施，同步开展招商引资，打造绿色食品加工、能源开发、旅游文化、商贸物流等跨境产业链，推动“大桥经济”发展。保税物流中心(B型)通过验收，推动升级为综合保税区。完善境外石化、农业、物流园区功能，吸引更多企业入驻。深化贸易投资合作。增强口岸功能，争取获批更多指定口岸资质，扩大机电、果蔬出口和电力、粮食、纸浆进口，挖掘能源、矿产、木材、绿色食品加工贸易和运输服务、医疗服务贸易潜力，巩固外贸增长态势。争取油品储运项目有新进展，阿州锑矿实现开采回运。扩大对俄农林合作规模，争取中俄跨境林业产业园项目落地，大豆低温浓缩豆粉一期投产。徐工欧亚汽车零部件生产项目开工，依托利源达覆盖俄全境的营销网络，打造集整车销售、配件生产于一体的装备制造基地。加强人文领域交流。高水平办好第十届中俄文化大集，打造中俄文化交流品牌。办好中俄建交70周年地方合作交流论坛、中俄边境城市展览会、中俄进出口商品订货会、中俄林业生态建设国际学术论坛、中俄界江国际冰球友谊赛、横渡黑龙江等重大活动，继续深化对俄科技、教育等领域交流，开展中俄美术、摄影、工艺品展览展销，促进情感交融，推动合作共赢。

(四)积极发展新产业新业态新模式，加快培育新的经济增长点。努力在符合发展方向、有需求增长支撑、黑河有供给能力的领域集中发力，促进现代服务业提档升级，实现发展动能转换。推动全域旅游发展。做好2020年全省旅游发展大会筹备工作，发挥五大连池风景区和市区两大核心带动作用，统筹农垦、森工旅游资源开发，推动观光式旅游向体验式旅游转变、夏季旅游向四季旅游转变、景区旅游向全域旅游转变。高标准编制全域旅游总体规划，按照5A级景区标准编制市区旅游规划，强化规划实施，实现景城一体。打造瑷珲乡野公园沿江风景带，推进夜游黑龙江项目常态化，加快黑河中俄民族风情园、北安博物馆群、五大连池朝阳山抗联红色教育基地、孙吴和逊克俄罗斯民族风情村等景区景点建设，嫩江墨尔根古道驿站博物馆、锦河大峡谷景区晋升国家3A级。统筹发展跨境游与内地游，深度开发赴俄旅游线路，积极开发自驾游、康养游、避暑游、生态游、研学游，扩大“黑河·我的极致远征”跨境自驾游品牌影响力，打造类型多样、广泛参与、覆盖四季的“狂欢节”。完善宾馆酒店、景区道路、自驾车营地、旅游厕所等配套设施，加强诚信建设，提升旅游形象。强化旅游市场监管，提高服务质量和标准。发展智慧旅游，推进黑河旅游景区数字化监控中心、五大连池智慧景区项目，实现4A级以上景区WIFI全覆盖，让游客一部手机游遍黑河。加快培育康养产业。推进五大连池风景区国际矿泉理疗城前期，加快高端医养康养项目建设，与省内外养老机构合作开发异地养老线路，打造定制式高端康养产品。鼓励民间资本投资康养产业，拓展跨国旅居养老互访体验活动范围，推广“候鸟式”养老、旅居养老、智能化养老模式，加快养老产业与旅游休闲、医疗卫生等产业融合发展。做大做强冰雪经济。高标准建设大黑河岛中俄冰雪乐园、五大连池风景区冬奥主题冰雪乐园，加快逊克大平台雾凇景区基础设施建设，开发五大连池山口湖雾凇景区。办好寒地试车旅游节、全民冰雪日、雾凇冰雪节等活动，谋划争取省级越野滑雪锦标赛落地黑河，开发极限驾驶、冰面漂移、雪地摩托、冬捕冬泳等体验式项目，把冷资源变成热经济。推进黑河寒区试车产业园建设，开工汽车实验基地、研发检测中心和试车大厦，打造国际试车产业基地。大力发展新兴服务业。扩大电商规模，依托瑷珲e购建设黑河生态产品新零售体验中心，地方特色产品上架电商平台，线上线下一体化销售。完善跨境电商园区功能，创建国家级电子商务示范基地。以金融创新助推现代服务业发展，积极开展跨境人民币结算业务，推动村镇银行等新型农村金融机构发展，创新产品供给，加强金融监管，有效防控金融风险。壮大珠宝玉石产业，扩大俄罗斯珠宝玉石加工规模，谋划建设黑河珠宝玉石展示中心，推进逊克玛瑙产业园二期，牵头制定北红玛瑙国家标准，叫响北红玛瑙品牌。

(五)加强生态保护和环境治理，巩固提升绿色发展优势。牢固树立生态文明建设首位意识，坚决守住生态保护红线、环境质量底线、资源利用上线。加快生态系统建设。推进“三北”防护林体系建设和中央财政补贴造林工程，完成造林绿化任务6万亩、森林抚育35万亩。发挥自然保护地生态功能优势，加大湿地资源保护与修复力度，统筹做好自然保护区、森林公园和湿地公园保护与管理。坚持四季抓防火，推进森林草原消防基础设施建设，完成260公里防火路修建任务，与森工、农垦做好联防联扑工作，坚决防止重特大森林草原火灾。强化污染综合防治。守护好“黑河蓝”，继续开展县级城市建成区10蒸吨及以下燃煤小锅炉淘汰工作，开工建设市热电厂环保节能技改项目，大力推进秸秆禁烧离田，加快凯迪、诺宝生物质发电等项目建设，提高秸秆综合利用率，让蓝天白云成为黑河最靓丽的名片。守护好清澈的水，严格落实河湖长制，强化饮用水水源地保护，加大水污染防治力度，开展好城镇供水水质、污水和垃圾治理能力提升三年行动，促进水环境持续改善，让清水绿岸常在。守护好肥沃的黑土地，农业“三减”实现全覆盖，加强对废弃农药瓶、农药袋回收管理，开展土壤污染状况和工业企业场地详查，对多宝山铜矿、三道湾子岩金矿、翠宏山铁多金属矿进行重点监控，防止重金属污染农田。全力推进中央环保督察“回头看”及农业农村污染专项督察反馈问题整改，确保完成年度整改任务。开展破坏生态环境问题“清零”专项行动，重拳打击破坏环境资源违法犯罪行为，加快还清生态环境欠账。推进绿色低碳发展。全面启动绿色矿山建设，生产煤矿全部建成“绿色煤矿”。加强对重点耗能企业节能监控，年耗能万吨以上规上企业全部纳入监测，严查违法用能、浪费能源行为，提高资源能源利用率。深入推进生态环保宣传进机关、进企业、进学校、进社区、进农村，推广绿色节能建筑，推行节地生态安葬，引导群众生活方式向绿色节约转变。

(六)提升城市品质形象，建设现代化生态口岸城市。以创建全国文明城市为抓手，推动城市硬件和软件同步改善，城市功能和品位同步提升，塑造别具特色的城市品牌形象。坚持精致规划。围绕打造中等以上规模城市，高标准推进新一轮至2035年城市总体规划修编。编制桥头区控详规划，完善口岸通道、商贸流通、生产加工、文旅休闲和体育等功能，推动老城区、产业区和新城区互动整合，打造高标准现代化新城区。严格执行大黑河岛改造规划，保护好绿色主基调，打造生态优美、产业繁荣、文化先进、活力迸发的生态旅游岛。加快推进“多规合一”，坚决维护规划权威性、严肃性。坚持精心建设。实施街路硬化工程，推进第四中学两侧断头路、龙海路、龙科街延伸段等道路建设，对兴安街等8条巷道和5处停车场进行维修改造，让群众出行“不添堵”。实施园林绿化工程，扩大冬季绿色树种栽植面积，绿化4条街路，建设8处五色草景观。实施楼体亮化工程，对市区重要节点楼体整体亮化，改造升级老旧亮化设施，用五彩灯光妆点城市。实施景观美化工程，完成瑷珲—腾冲人口地理分界线主题公园主体工程，改造升级世纪广场，新增街角城市小品，以城市摄影师的视角，结合自然风光和标志性建筑，建设若干个城市观景平台。完善小区配套设施，已建小区尽快补齐服务设施短板，新建小区同步配建社区综合用房和群众活动场所。完成市区126栋楼体立面维护，维修改造90栋老旧住宅楼，加强老旧小区物业管理、弃管楼综合管理，推进老旧楼房电梯改造试点，有计划分步骤实施二次供水升级改造。完善棚改项目配套设施，确保居民如期回迁入住。推进城市燃气工程，新增覆盖居民1.5万户。打造魅力县城，加快北安祥和公园三期、嫩江生态景观公园、五大连池城区防洪堤景观带、逊克滨江公园、孙吴建华桥公园建设，让每个县城更有颜值。坚持精细管理。开展城市精细化管理提升年活动，用“绣花功夫”“工匠精神”管理城市，完善环卫作业指标体系，加大市政设施精细化管护力度，集中整治主要街路违法建设、占道经营等城市乱象，建立市区一体管理模式，让城市更有序、更干净、更秀美。推进智慧城市建设，启动数字城管平台升级改造和智慧城管网络系统项目，推进市县两级数字化平台技术整合对接，利用大数据实现城市管理智能化。

(七)更加关注补齐民生短板，让群众共享振兴发展成果。坚持群众关注什么，我们就抓什么；群众不满意什么，我们就改进什么，尽心尽力为群众办好事、办实事。全力打赢精准脱贫攻坚战。坚持目标不变、靶心不散、频道不换，咬定总攻目标，一鼓作气、再接再厉，全力打赢决胜之战，确保现行标准下贫困村、贫困人口全部脱贫摘帽。坚持产业扶贫主攻方向，着力建设一批贫困人口参与度高、持续增收强的乡村特色产业。充分发挥扶贫小额信贷资金作用，提高扶贫资金拨付进度和使用效率，当年扶贫资金拨付率达到95%以上。完善脱贫攻坚长效机制，制定脱贫退出巩固提升方案，实施后续帮扶计划，严控返贫率，确保脱贫成果经得起检验。办好人民满意教育。深化教育体制机制和新时代教师队伍建设改革，推广嫩江义务教育学校教师“县管校聘”管理改革经验，与省同步启动高考综合改革，全市新建改扩建公办幼儿园3所，北安、嫩江、五大连池(含风景区)义务教育基本均衡发展通过国检，深化职业教育校企合作，成立黑河社区大学，提升老年大学办学水平，办好黑河技师学院。加快建设“健康黑河”。加大公共卫生投入力度，加强重大传染疾病防控。深化医药卫生体制改革，加快建立现代医院管理制度，抓好嫩江第一人民医院薪酬制度改革试点，严格控制全市公立医院医疗费用增长幅度，降低17种抗癌药品价格并纳入医保目录，五大连池人民医院康复中心综合楼投入使用，北安启动申报国家卫生城市。加强中俄卫生交流，深化诊疗技术合作。打造边疆特色文化体育品牌。办好市民社区文化节等群众文化活动，组织创作优秀文艺作品，扩建升级黑河俄罗斯油画城，筹建黑河图书馆和史志档案馆，谋划建设黑河地名文化馆、科技馆，加强博物馆建设，弘扬独具特色的北疆文化。举办市第六届运动会，开展“冰雪体育之冬”“百万青少年上冰雪”系列活动，启动全国全民运动健身模范市(县、市、区)创建工作，推动建设黑河冰球馆，新建冰雪类体育场地170处，形成15分钟健身圈，打造充满活力的动感城市。推进人才强市建设。用心用情用事业用待遇留住人才，制定出台更有含金量的人才政策，大力培养选拔本地人才，柔性引进高层次人才，为研究生、一本院校毕业生提供人才绿色通道，深化对俄人才交流合作，让各类人才创业有机会、干事有舞台、发展有空间。完善就业和社会保障体系。以创新引领创业、创业带动就业，推进各类孵化服务机构发展和科技型企业孵化培育，促进型支出占就业资金支出比重提高到50%，全市新增就业1.45万人，城镇登记失业率控制在4.3%以内。加大社会保险扩面征缴力度，推进机关事业单位养老保险制度改革。完善城乡基本医疗保险制度和大病保险制度，实施以按病种付费为主的多元复合式医保支付方式改革，稳步推进医保异地就医直接结算，城乡居民医疗保险政府补贴标准由每人每年490元提高到520元。提高贫困群体综合救助和精准救助能力，推进养老院服务质量标准化建设，完成北安、逊克、爱辉区中心敬老院适老化改造，让更多老年人安享幸福晚年。大力发展慈善事业，建立政府推动、社会支持、群众参与的新机制。维护社会和谐稳定。提升社区服务功能，推进城乡社区治理体系建设。完善安全生产和应急管理责任体系，健全风险防控和隐患排查治理双重预防机制，深入推进“智慧消防”建设，加快应急管理信息化建设，提高应急管理保障能力，全面防范遏制事故灾害，坚决遏制重特大安全生产事故发生。坚持属地属事相结合，深入开展社会矛盾纠纷排查化解专项行动，领导干部带头解决疑难信访问题，维护群众合法利益诉求。开展公共安全隐患排查治理行动，完成城乡视频监控“雪亮工程”建设，高压纵深推进扫黑除恶专项斗争，推进社会治理体系现代化，提高预测预警预防能力，打造“平安黑河”升级版。支持国防建设和军队改革，巩固全国双拥模范城创建成果，推进军民融合深度发展，维护边境安全稳定。依托现有公园建立国防教育主题公园。落实好退役军人接受安置、抚恤、优待、培训等保障政策，维护退役军人合法权益。进一步做好老区、侨务、妇女儿童、民族宗教等工作。

(八)重塑营商环境，用良好环境聚人聚财聚发展要素。深入贯彻落实省优化营商环境条例和重塑营商新环境的意见，以“办事不求人”为目标，着力打造高效透明的政务环境、公平正义的法治环境、诚信守约的人文环境、绿水青山的生态环境、风清气正的政治生态环境，形成“人人都是营商环境、个个都要做好黑河主人”的浓厚氛围。推进“放管服”改革。以“放”为原则，以“不放”为特例，让企业和群众办事“只进一扇门”。加快全市一体化在线政务服务平台建设，实现政务服务“一张网”。推进“最多跑一次”改革，做到大多数事项“网上办”，其他事项网上预约“现场办”，必须到窗口事项“一次办”，企业特殊审批事项“我帮办”。探索实行企业投资项目“承诺制、零审批”改革和政府投资项目“一表一窗”联审会审，对重点项目实施“代办制”“驻场制”，以“保姆式”服务定期跟踪项目进展、手续办理、完成投资等情况。制定出台公共资源交易管理办法和交易目录，整合纳入统一平台，实现阳光交易。降低企业制度性交易成本。推行多证合一、证照分离，推进市场主体登记全程电子化，企业开办时间压缩到8个工作日以内，不动产登记5个工作日内办结。落实便利办税措施，提升纳税服务水平。深入推进“双随机一公开”监管模式，加强事中事后监管，开展营商环境评价。构建诚信体系，健全诚信“黑名单”制度，严厉查处失信违诺行为。激发市场主体活力。全面深化国资国企改革，建立现代企业制度，抓好市场化选聘职业经理人试点，以资金加平台模式吸引社会资本，确保国有资产保值增值。认真落实国家和省扶持民营经济发展政策，制定出台具体措施，增强民营经济活力。深入开展质量提升行动，各县(市、区)全部设立政府质量奖。建立完善挂牌上市企业后备资源库，支持具备条件企业上市融资。建立市级协调推进机制，定期举办政银企对接会，加强金融服务实体经济情况评估，积极帮助企业解决难题，增强亲商尊商富商意识，构建“亲”“清”新型政商关系。

三、全面加强政府自身建设

使命重在担当，实干铸就辉煌。当前，黑河发展改革开放的蓝图已经绘就，目标已经明确。我们要敢于刀刃向内自我革命，在敢为上勇担当，在能为上增才干，在有为上重实效，不断提升履职能力和水平。旗帜鲜明讲政治。扎实开展“不忘初心、牢记使命”主题教育，树牢“四个意识”，坚定“四个自信”，做到“两个维护”，增强政治敏感性，站稳政治立场，铸牢忠诚“第一底色”。强化风险意识，保持斗争精神，全面防范化解政治、意识形态、经济、科技、社会、外部环境、党的建设等领域重大风险，应对好每一场重大风险挑战。对于中央和省重大决策部署，用最快的时间反应、最短的期限落实、最好的成效反馈，坚决杜绝视而不见、反应迟钝、漠然处之、无动于衷。打开解放思想“总开关”。以解放思想推动高质量发展大讨论为契机，着力破除传统思维观念束缚，坚决向“旧、怕、平、懒”突出问题宣战。要有想干事、能干事、干成事的气魄，只要有利于深化供给侧结构性改革，有利于加快培育经济增长新动能，有利于激发市场主体活力，有利于增强人民群众获得感，有利于调动广大干部积极性，就要敢想敢干、敢闯敢试、敢打敢拼，把解放思想的成效体现到推动高质量发展上。提高依法行政水平。树立法治思维，坚持依法办事，加强立法工作，把政府工作全面纳入法治化轨道。坚决贯彻市委各项部署，自觉接受人大依法监督、政协民主监督，广泛听取社会各界意见和建议，确保政府工作更加符合社情民意、符合法治要求。严格执行重大行政决策程序，规范以政府名义签订的协议和契约，新官也要依法依规理旧账。全面落实“七五”普法规划，增加学法频次，提高学法质量，强化学法考核，以政府尊法知法带动全社会信法守法。弘扬务实担当作风。从小事小节做起，从文风会风改起，改出新气象、新担当、新作为。加强机构改革后政府系统履职尽责意识和素质能力建设，探索实施政府绩效管理，实行扁平化模式，把继承优良传统与科学方法结合起来，给“良心活”套上“硬约束”，让“旁观者”变成“干事者”。大力倡导走上去、走出去、走下去，少些痕迹、多些足迹，不搞花架子、不做表面文章，抓重点抓关键，精准整治形式主义官僚主义，不让职责空位，不使工作空转，提振精气神，时刻在状态，把全部精力用在抓落实上。守住廉洁从政底线。认真履行全面从严治党主体责任，严格落实“一岗双责”，既紧盯“关键少数”，又管好大多数，以零容忍的态度坚决惩治腐败，做到真管真严、敢管敢严、长管长严。牢固树立过“紧日子”思想，从严控制“三公”支出，用政府的“紧日子”换来群众的“好日子”。充分发挥审计监督作用，加强重点资金和项目审计监管，强化权力运行监督制约，树立政府清廉清正清明良好形象。

各位代表，“新时代是奋斗者的时代”，“幸福都是奋斗出来的”。让我们更加紧密地团结在以习近平同志为核心的党中央周围，在省委、省政府和市委的坚强领导下，解放思想、振奋精神，以一往无前的奋斗姿态，书写黑河新时代改革开放新篇章，以经济社会发展的新成果，胜利迎接中华人民共和国成立70周年！
